# Digital Clinical Photography: Practical Tips

**DOI:** 10.4103/0974-2077.63285

**Published:** 2010

**Authors:** Sharad Mutalik

**Affiliations:** *Consultant Dermatologist, Cutaneous and Laser Surgeon, Planet Skin, Pune, Maharashtra, India*

**Keywords:** Digital camera, pixel, zoom, sensor

## Abstract

Photographs are the most preferred and easiest way of documentation of patient visual features. In aesthetic and cutaneous surgery, there is an increased need for proper photographic documentation, from a medicolegal view point. This article discusses the basic aspects of camera and photography which a dermatologist should be aware before he/she starts with clinical photography.

## INTRODUCTION

Digital photography is a form of photography that uses digital technology to make images of subjects. Until the advent of such technology, photographic films were used to create images which could be made visible by photographic processing. In contrast, digital photographs can be displayed, printed, stored, manipulated, transmitted and archived using digital and computer techniques, without chemical processing. Digital photography has become hugely popular and has been adopted by amateur snapshot photographers, as it is very convenient and user friendly. It is an important and a useful asset for record keeping and can be used as evidence in clinical practice, especially dermatology.[1,2] This article describes the salient aspects of digital photography.

## ADVANTAGES OF DIGITAL PHOTOGRAPHY

1. It is possible to see digital images immediately. If captured photographs are not satisfactory, another picture can be clicked in a different setting.
2. Photographs can be sent instantly through e-mail/mobile telephone or printed and shared, seconds after taking them. Pictures can be uploaded on World Wide Web. Thus, it is a useful tool for telemedicine.
3. Photographs can be corrected, changed, improved, to make the pictures better with ‘digital magic’. One can use picture-editing software to lighten pictures, get rid of red eye, crop pictures, and make other improvements after they were taken.

## DEFINITIONS: TERMS USED IN DIGITAL PHOTOGRAPHY

It is important to get acquainted with some terms in digital photography, before one starts using a digital camera; these include pixel, resolution, optical zoom and JPEG.[3,4]

### Pixel

A pixel is a contraction of the term *pi*cture *el* ement. Pixel is the smallest element of a digitized image. Digital images are made up of small squares, just like a tile mosaic on a kitchen or bathroom wall. Though a digital photograph looks smooth and continuous just like a regular photograph, it is actually composed of millions of tiny squares or pixels. Pixels are also defined as total pixels and effective pixels (see below).

*Pixel count*: The number of pixels *n* (number) for a given maximum resolution (*w* horizontal pixels by *h* vertical pixels) is the product *n* = *w × h*.

For example, for a picture having 1600 pixels in width and 1200 pixels in height, the resolution or pixel count is 1.92 megapixels, 1600 (*w*) × 1200 (*h*) = 1,920,000 (*n*) = 1.92 megapixels.

Digital cameras are categorized in terms of pixel count. It is the total number of individual pixels that go into making each image. In the cameras available now, this number varies from 1 million (1 megapixel) to around 14 million (14 megapixels). The term ‘million pixels’ is abbreviated to MP, so a 1 MP camera has 1 million pixels and a 3 MP camera has 3 million pixels. Currently, most commonly used consumer digital cameras have between 2 MP and 12 MP. A 3 MP camera can make excellent 4″×6″ prints and very good 5″×7″ prints [Table 1]. If 8″×10″ or bigger prints are needed, then perhaps a 4 MP or 5 MP camera would be a better choice.

**Table 1:** Number of pictures stored on the card depending upon the memory of the card and megapixels

Card size (MB)	4 MP	5 MP
64	26	18
128	52	37
256	104	74

### Resolution

Resolution provides an indication of the amount of detail that is captured. But, like the other metrics, resolution is just another factor out of many in determining the quality of an image. A higher number of pixels in an image correlates to a higher resolution. The higher the resolution, the more pixels in an image and therefore the greater image quality.

### Sensor

Sensor is the part that records the image and is always expressed in megapixels. The greater the number of megapixels, the more information sensor can capture and the more an image can be enlarged. Total pixels indicate every pixel on the sensor surface. However, the very edge pixels are not used in the final image. Effective pixels are the number of pixels actually used in the image after the edge pixels have been dropped.

### Zoom control

In many situations, one may want to get ‘close’ to a subject without moving physically closer. The zoom lens serves this purpose. The zoom control lets you get ‘close’ enough to capture that image.

Most cameras have both optical zoom and digital zoom. Optical zoom works just like a zoom lens on a film camera. The lens changes focal length and magnification as it is zoomed. Image quality stays high throughout the zoom range.

An optical zoom lens can extend physically to magnify the subject.

The image quality of the picture captured through an optical zoom lens stays high throughout the zoom range. Optical zoom allows you to get closer to your subject when you want to be discreet about taking pictures, like at a ceremony or function.

Digital zoom, on the other hand, simply crops the image to a smaller size, and then enlarges the cropped portion to fill the frame again (interpolation). Digital zoom results in a significant loss of quality as is clear from the examples below and therefore it's usually a last resort. Any image editing program can do similar digital manipulation. Most of the mobile cameras have digital zoom lenses.

Because of the small, poor quality lenses and sensors in most of the cell phones, the quality of the pictures makes them unsuitable for making even moderate size prints. However, capturing the moment, though of lesser quality is better than not getting the picture at all. For example, if an interesting case comes and in the absence of a proper camera, there is no other choice but to capture the image in the cell phone camera.

## CAMERA SETTINGS

Each digicam has following settings: auto/program/p, flash on/off, macro, video, landscape, sports, night landscape, and night portrait. For clinical photography, we need to use only the macro mode. Some cameras have an additional inbuilt digital image stabilization technology (blur reduction technology). It reduces blur caused by camera shake, subject movement, or fast action situations.

### Points to remember in order to capture a clear image

The following aspects should be carefully considered to capture a clear image: adequate lighting in the room and position of light, patient's position, background and distance of camera from the patient:[3,4]

**Figure 1:**
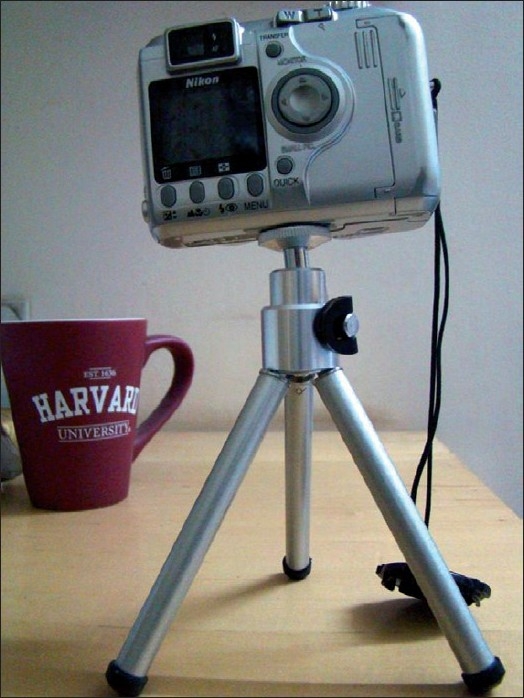
Camera on a tripod

1. It is always preferable to take pictures in natural diffuse sunlight. The patient must face the slanting sun rays, so as to get a shadowless picture. For indoor photography, white fluorescent diffuse light should be used.
2. Stand at a distance of 3 ft from the patient, set the camera on macro mode, and flash off, and make sure that no shadow or glare is seen in the view finder; zoom in and shoot.
3. When you press the switch ‘W’ or ‘T’ on the camera, the subject is either magnified or reduced in size. ‘W’ stands for ‘wide angle’ (reduce) and ‘T’ stands for ‘telephoto’ (magnify). Use a tripod to avoid camera shake [Figure 1]. If a tripod is not available, give chin rest to the patient [Figure 2].

**Figure 2:**
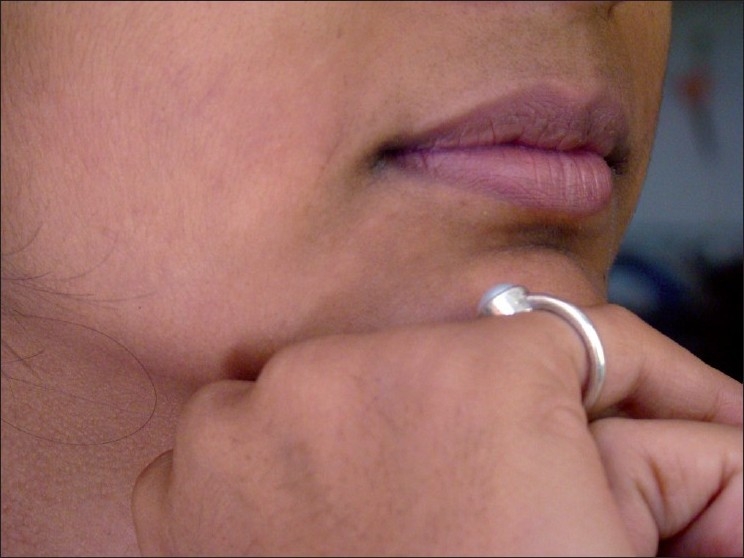
Chin on a rest
4. Use green or bottle green background.
5. Place a sticker label of patient's reference number on the part to be picturized, so that it becomes easier to locate the patient and his or her photographic record. Make a note of the position of the patient (front, profile, dorsum, ventral, etc.) on the clinical records. It is easier to take the follow-up clinical photo in the same position with same lighting and background.

### How to focus and shoot

Before taking the actual picture, a camera needs to focus, work out exposure, to be ready to record the image. The time taken from pressing the shutter release to taking the picture is called *shutter lag*.

It is a problem in many compact cameras and even more in digital cameras. This delay is different in different cameras and one should check it in store before buying it as a long shutter lag will miss the ‘decisive moment’, as an object may already have moved out of the frame by the time the picture is taken.

The way to minimize this with any camera is pre-focusing. When the shutter release is pressed halfway, the camera will focus, set exposure and if needed charge the flash, indicating in the display or viewfinder when it is ready. Keeping the shutter depressed halfway until the best moment and then pressing it fully allows the camera to take the picture almost instantly; if it does not, move on to the next model.

Macro mode is the best mode to capture image in exquisite detail, such as the lines in a baby's tiny hand, creases, papules, vesicles, etc.[5–7]

### Transferring and saving the pictures

Transfer your pictures to the personal computer (PC) through a card reader/USB cord. Set pictures into two folders: family photos and clinical photos. A standardized format used for storing images which is one of the most widely used formats today is *Joint Photographic Experts Group* (JPEG).[8] It is commonly used for images on the web and images attached to e-mail messages. JPEG, the group that established this file standard, is a standardized image compression mechanism.

In the clinical photographs folder, subfolders can be made according to the diagnosis and classified. The PC automatically arranges these folders alphabetically.

Enter the diagnosis, patient's initials and reference number in the individual picture's file name, e.g. psoriasis scalp Mr. DGM 09/2345 occiput before treatment. Make it a habit to transfer the images at least fortnightly if not weekly, when your memory is fresh about the patient.

Always having a back-up on a portable hard drive is highly essential. If anything goes wrong with hard disk of the camera, valuable records are lost. So it is always advisable to keep a back-up on a portable hard drive or pen drive.

### Which camera to buy?

Before buying any camera, one must decide what he/she want to get out of a digital camera. On most websites as well as a shop's shelves, cameras are lined up by their ‘megapixel’ count. Print sizes are given in 300 and 150 PPI, or pixels per inch. 300 is generally regarded as the optimal resolution, but bigger prints tend to be viewed from further away and one can therefore usually get away with a lower PPI count. Ideally one should go for a digital camera with a macro mode, with a good optical zoom, and midway between a compact and SLR digital camera (Prosumer), which is light in weight but serves the purpose of clinical photography in dermatology. Information on consumer cameras of all the established brands is available on WorldWideWeb and one can compare all the models before finalizing and buying.

## SUMMARY

The advantages of digital photography over the traditional film include the following:

1. Instant review of pictures, with no wait for the film to be developed. If there's a problem with a picture, the photographer can immediately correct the problem and take another picture.
2. Minimal ongoing costs for those wishing to capture hundreds of photographs for digital uses, such as computer storage and e-mailing.
3. Photos may be copied from one digital medium to another without any degradation.
4. Ability to print photos using a computer and consumer-grade printer.
5. Cameras can ‘imprint’ a date over a picture by exposing the film to an internal LED array (or other device) which displays the date.
6. Ability to capture and store hundreds of photographs on the same media device within the digital camera; in contrast, a film camera would require regular changing of the film (typically after every 24 or 36 shots).
7. Many digital cameras now include an AV-out connector (and cable) to allow the reviewing of photographs to an audience using a television.
8. Anti-shake functionality (increasingly common in inexpensive cameras) allows taking sharper hand-held pictures where previously a tripod was required.
9. Smaller sensor format, compared to a 35 mm film frame, allows for smaller lenses, wider zoom ranges and greater depth of field.
10. Ability to use the same device to capture video as well as still images.
11. Ability to convert the same photo from colour to sepia to black and white.
